# Synergism between the phosphatidylinositol 3-kinase p110β isoform inhibitor AZD6482 and the mixed lineage kinase 3 inhibitor URMC-099 on the blockade of glioblastoma cell motility and focal adhesion formation

**DOI:** 10.1186/s12935-020-01728-4

**Published:** 2021-01-06

**Authors:** Hua-fu Zhao, Chang-peng Wu, Xiu-ming Zhou, Peng-yu Diao, Yan-wen Xu, Jing Liu, Jing Wang, Xian-jian Huang, Wen-lan Liu, Zhong-ping Chen, Guo-dong Huang, Wei-ping Li

**Affiliations:** 1 Department of Neurosurgery, Shenzhen Second People’s Hospital/the First Affiliated Hospital of Shenzhen University Health Science Center, Shenzhen, 518035 China; 2 Department of Neurosurgery, People’s Hospital of Longhua District, Shenzhen, 518109 China; 3 Epilepsy Center, Guangdong 999 Brain Hospital, Guangzhou, 510510 China; 4 Department of Neurosurgery/Neuro-Oncology, Sun Yat-Sen University Cancer Center, State Key Laboratory of Oncology in South China, Collaborative Innovation Center for Cancer Medicine, Guangzhou, 510060 China

**Keywords:** Glioblastoma, PI3K, p110β, MLK3, Synergism

## Abstract

**Background:**

Glioblastoma multiforme, the most aggressive and malignant primary brain tumor, is characterized by rapid growth and extensive infiltration to neighboring normal brain parenchyma. Our previous studies delineated a crosstalk between PI3K/Akt and JNK signaling pathways, and a moderate anti-glioblastoma synergism caused by the combined inhibition of PI3K p110β (PI3Kβ) isoform and JNK. However, this combination strategy is not potent enough. MLK3, an upstream regulator of ERK and JNK, may replace JNK to exert stronger synergism with PI3Kβ.

**Methods:**

To develop a new combination strategy with stronger synergism, the expression pattern and roles of MLK3 in glioblastoma patient’s specimens and cell lines were firstly investigated. Then glioblastoma cells and xenografts in nude mice were treated with the PI3Kβ inhibitor AZD6482 and the MLK3 inhibitor URMC-099 alone or in combination to evaluate their combination effects on tumor cell growth and motility. The combination effects on cytoskeletal structures such as lamellipodia and focal adhesions were also evaluated.

**Results:**

MLK3 protein was overexpressed in both newly diagnosed and relapsing glioblastoma patients’ specimens. Silencing of MLK3 using siRNA duplexes significantly suppressed migration and invasion, but promoted attachment of glioblastoma cells. Combined inhibition of PI3Kβ and MLK3 exhibited synergistic inhibitory effects on glioblastoma cell proliferation, migration and invasion, as well as the formation of lamellipodia and focal adhesions. Furthermore, combination of AZD6482 and URMC-099 effectively decreased glioblastoma xenograft growth in nude mice. Glioblastoma cells treated with this drug combination showed reduced phosphorylation of Akt and ERK, and decreased protein expression of ROCK2 and Zyxin.

**Conclusion:**

Taken together, combination of AZD6482 and URMC-099 showed strong synergistic anti-tumor effects on glioblastoma in vitro and in vivo. Our findings suggest that combined inhibition of PI3Kβ and MLK3 may serve as an attractive therapeutic approach for glioblastoma multiforme.

## Background

Glioblastoma multiforme (glioblastoma, GBM), the highest grade of glioma (grade IV) in the World Health Organization (WHO) classification, is the most common and the most malignant aggressive primary brain tumor with a high mortality rate [1, 2]. Phosphatidylinositol 3-kinases (PI3Ks) are lipid kinases involved in diverse biological responses including cell proliferation, glucose metabolism, differentiation, motility and angiogenesis [3, 4]. Although dysregulation of PI3K/Akt signaling pathway is common in GBM, blockade of its activation by specific PI3K inhibitors is not an effective therapeutic approach for GBM treatment [5, 6]. Generally, blockade of PI3K/Akt signaling could give rise to the compensatory activation of other pathways such as the mitogen-activated protein kinase (MAPK) pathway, relieving the inhibitory effects and maintaining tumor cell survival [7, 8]. Therefore, combination therapies by targeting PI3K and other molecules may be an effective approach for GBM treatment and facilitate better benefit to patients. The c-Jun N-terminal kinase (JNK) signaling, a branch of MAPK signaling, plays a pivot role in the survival and motility of cancer cells and has a crosstalk with PI3K/Akt signaling [9]. Our previous study showed that combination of the selective PI3K p110β isoform (PI3Kβ) inhibitor (TGX-221) and the JNK inhibitor (SP600125) displayed moderate synergistic inhibitory effects on *in-vitro* GBM cell proliferation, migration and *in-vivo* xenograft growth [10]. However, this synergism was not potent enough to inhibit GBM cell invasion, and the tumor suppressive effect was mild. Therefore, to improve the efficacy of this synergism on GBM, development of more potent combination strategies is urgently needed.

Mixed lineage kinases (MLKs) belong to a large family of mitogen-activated protein kinase kinase kinase (MAP3K) and are consist of three subfamilies: MLKs, the dual leucine zipper-bearing kinases (DLKs), and the zipper sterile-α motif kinases (ZAKs). MLK3, a member of MLKs subfamily, is a functional serine/threonine protein kinase that contributes to the activation of MAPKs including JNK, p38, and extracellular signal-regulated kinase (ERK) [11, 12]. Activated ERK and JNK could translocate to the nucleus, phosphorylate and interact with the transcription factors such as c-Jun, c-myc and c-fos, leading to the regulation of various cellular progresses [13]. Accumulating studies show that MLK3 is involved in the regulation of cell tumorigenesis, migration and invasion of cancers [14–16]. Due to the crosstalk and constitutive activation of PI3K/Akt and Ras/MEK/ERK signaling in GBM, dual inhibition of these two pathways display synergistic anti-glioma effects in vitro and in vivo [17–19]. Also, this combination strategy has entered into clinical trials for patients with advanced solid tumors [20, 21]. Considering that blockade of MLK3 could inhibit both JNK and ERK, combined inhibition of PI3Kβ and MLK3 may display stronger synergistic anti-glioblastoma effects, compared with dual inhibition of PI3Kβ and JNK, or PI3Kβ and ERK.

In this study, we showed that high expression of MLK3 was found in tumor tissues from both newly diagnosed and relapsing GBM patients. Silencing of MLK3 significantly suppressed the migration and invasion, but promoted the adhesion of GBM cells. Using the isoform-selective PI3Kβ inhibitor AZD6482 and the broad-spectrum MLK3 inhibitor URMC-099, concurrent inhibition of PI3Kβ and MLK3 showed synergistic inhibitory effects on the proliferation, migration and invasion of GBM cells. Combination of AZD6482 and URMC-099 exerted synergism on impeding the formation of lamellipodia and focal adhesions (FAs). It also reduced the phosphorylation of Akt and ERK, and the protein expression of Rho-associated protein kinase 2 (ROCK2) and Zyxin. Furthermore, this drug combination also effectively decreased GBM xenograft growth in nude mice. These results suggested that combined inhibition of PI3Kβ and MLK3 may be a more effective combination strategy for GBM treatment.

## Materials and methods

### Cell culture

Glioblastoma cell lines (including U-87 MG, U-118 MG, U-138 MG, U-343 MG, U-373 MG, U-251 MG, A-172, LN-Z308 and SK-MG3) and normal human astrocytes were cultured as described previously [10]. U-87 MG, U-118 MG, A-172 and U-251 MG were obtained from the Cell Bank of Chinese Academy of Sciences. U-138 MG, U-343 MG, U-373 MG, LN-Z308 and SK-MG3 were kindly provided by Dr. Shing-shun Tony To (The Hong Kong Polytechnic University). Normal human astrocytes cell line was purchased from ScienCell Research Laboratories. Cells were cultured in the minimum essential medium alpha (α-MEM) supplemented with 10% (v/v) fetal bovine serum (FBS) (Cat.No 10099, Thermo Scientific). Cells were incubated at 37 °C in 5% CO_2_ atmosphere.

### Tumor specimens

Tumor specimens were retrospectively obtained from 47 patients with average age of 45.2 years (range from 19 to 74 years) who were histologically diagnosed as GBM (WHO grade IV) in the Shenzhen Second People’s Hospital from Aug 2012 to Dec 2016. Among the 47 GBM patients, there are 37 patients with newly diagnosed GBM and 10 patients with relapsing GBM. 2 grade I gliomas (including 1 angiocentric glioma, 1 pilocytic astrocytoma) and 2 gliosis were used as negative control. All samples were reviewed by an experienced pathologist. This study was approved by the Research Ethics Committee of Shenzhen Second People’s Hospital. All patients were given written informed consent.

### Chemical compounds

The MLK3 inhibitor URMC-099, the PI3Kβ inhibitor AZD6482, the pan-PI3K inhibitor BKM120 and the dual PI3K/mTOR inhibitor PQR309 were purchased from Selleckchem. The characteristics of these inhibitors were showed in the Additional file 1: Tables S1 and S2.

### Quantitative real-time PCR (qRT-PCR)

Total RNAs of GBM tissues were isolated using the TRIzol Reagent (Thermo Scientific) and the first-strand cDNAs were synthesized from total RNAs using the Fermentas RevertAid First Strand cDNA Synthesis Kit (Thermo Scientific). Primers of *MLK3 (MAP3K11)* and *GAPDH* genes for SYBR Green qRT-PCR were designed using Primer Premier 6 Software. Their sequences were as below. *GADPH*: forward primer, ATGGCACCGTCAAGGCTGAGAA; reverse primer, TGCTGATGATCTTGAGGCTGTTGTC. *MLK3*: forward primer, AACCTGTGCCTGGTGATGGAGTAT; reverse primer, GTTGGACTTGAGATCACGGTGGATG. The SYBR Green qRT-PCR was performed on ABI Quantstudio™ DX using the FastStart Universal SYBR Green Master Mix (Roche). According to the manufacturer’s protocol, the PCR reaction mixture contained 1 × SYBR Green Master Mix, template cDNA (0.05 μg) and primer pairs (0.3 μM). Human GAPDH mRNA was served as an internal control for RNA normalization. The relative expression was normalized using the 2^−ΔΔ^CT method. Independent experiments were carried out in triplicate, and each reaction is duplicated.

### RNA interference

Small interfering RNA (siRNA) synthetic duplexes targeting *MLK3* (*MAP3K11*) gene were purchased from Qiagen (Cat.No: SI00605626 and SI02659552) and appropriate RNase-free water was added to obtain a 20 μM solution. The AllStars Negative Control siRNA (Qiagen) with sequence not homologous to any known mammalian genes was used as a negative control. Briefly, GBM cells were seeded onto 6-well plates (1.5 × 10^5^ cells per well) and transfected with siRNA duplexes (100 pmol) using Lipofectamine 2000 transfection reagent (Thermo Scientific) according to manufacturer’s instructions. Cells were then incubated at 37 °C for further analysis.

### Cell proliferation assay (MTT method)

As described before [10], 2,000 GBM cells were seeded onto the each well of 96-well plates, and then were treated with inhibitors for 48 h. The 3-(4,5-Dimethylthiazol-2-yl)-2,5-diphenyltetrazolium bromide (MTT) was added, followed by incubation for 4 h at 37 °C. The formazan crystal was subsequently dissolved in 150 μL of dimethyl sulfoxide (DMSO). Absorbance at 570 nm was determined by Multiskan GO microplate spectrophotometer (Thermo Scientific). As described by Chou, combination effect of two drugs was evaluated by the combination index (CI) [22]. Fraction affected (Fa), referring to the inhibition of cell proliferation, was calculated as followed: Fa = 1- (% cell proliferation/100). According to the Fa values, CI was calculated by Compusyn software (CI indications: CI < 0.9: synergistic effect; CI > 1.1: antagonistic effect; 0.9 ≤ CI ≤ 1.1: additive effect). Experiments were carried out for three times, and each independent experiment consisted of four replicates.

### Cell attachment assay

A 96-well plate was pre-coated with 50 μg/mL Matrigel matrix (Corning) at 37 °C for 1 hr. After aspiration, 10 mg/mL heat-denatured bovine serum albumin (BSA) was subsequently added and incubated for 30 min to block the remaining sites which was not covered by Matrigel. Wells blocked by heat-denatured BSA without pre-coated Matrigel were used as the blank control. Cells (5 × 10^4^ cells per well) were seeded onto 96-well plates, and then incubated at 37 °C for 30 min to allow cell attachment. Subsequently, nonadherent and loosely attached cells cells were washed away by phosphate-buffered saline (PBS) for three times. The relative number of remaining cells was determined by MTT assay as described above. Experiments were carried out for three times, and each independent experiment consisted of four replicates.

### Wound healing assay

As described before [10], GBM cells (3 × 10^5^ cells per well) were seeded onto 12-well plates and cultured to 90%-100% confluence. Cells were then pretreated with 5 μg/mL mitomycin C for 1 h to eliminate the interference of cell proliferation. Wounds were produced using a sterile 200 μL pipette tip. After rinsed with PBS for three times, cells were photographed immediately (time zero) and at 24 h after wounding. The number of cells migrating into the original wounds was counted. Migration rate was referred to the number of cells migrating into the original wounds. Experiments were carried out for three times, and each independent experiment consisted of two replicates.

### Immunofluoresescence staining

As described before [10], GBM cells (2.5 × 10^4^ cells per well) grown on sterile coverslips in 24-well plates were fixed with 4% paraformaldehyde, permeabilized with 0.25% Triton X-100, and then blocked with 1% BSA. Cells were then incubated with appropriate primary antibody overnight at 4 °C, followed by Alexa Fluor-conjugated secondary antibody at room temperature in the dark. Actin filaments (F-actin) were then stained with CytoPainter phalloidin-iFluor 555 reagent (Abcam), microtubules were stained with a monoclonal antibody against α-tubulin, and nuclei were stained with DAPI. Focal adhesions were visualized using monoclonal antibodies against focal adhesion markers such as vinculin and paxillin. Coverslips were air dried and mounted with ProLong Gold antifade mountant (Thermo Scientific). Images were acquired using Leica TSC SP8 confocal laser scanning microscope. In each case, about 50 cells were photographed and representative cells were shown. Independent experiments were carried out in triplicate.

### Boyden chamber migration and invasion assays

GBM cells (2.5 × 10^4^ cells per insert) treated with inhibitors were seeded into the polyester membrane transwell inserts with 8 μm pores (Corning), and then the inserts were placed in the companion 24-well plates. For migration assay, the inserts maintained uncoated, whereas the inserts were pre-coated with Matrigel (2–3 ng) for invasion assay. α-MEM medium supplemented with 5% FBS in the companion plate was served as a chemoattractant. Cells were incubated for appropriate time at 37 °C (6 h for migration assay and 24 h for invasion assay). Non-transmigrated cells were removed by cotton swabs, while cells transmigrated to the lower surface of the membrane were fixed with absolute methanol and stained with 0.1% crystal violet solution. Cells were photographed under a light microscope at 40 × magnification and cells from at least 5 representative fields were counted using Image-Pro Plus software. Independent experiments were carried out in triplicate.

### Tumor xenograft in vivo

GBM cells (5 × 10^6^ cells) were subcutaneously injected into the lower flanks of 4-week-old Balb/C nude mice (female, 16–20 g, Vital River Laboratories). Once the tumors reached an average volume of approximate 70–80 mm^3^, mice (n = 6) of each group were intraperitoneally injected once daily for 7 days with vehicle [10% DMSO (v/v), 30% polyethylene glycol 400 (v/v), 1% Tween 80 (v/v) and 59% saline (v/v)], AZD6482 (30 mg/kg), URMC-099 (3 mg/kg), and combination of these two drugs. Tumor volumes were determined by caliper measurement every 2–3 days as followed: Volume = (width)^2^ × length/2. Length is the longest diameter and width is the shortest diameter perpendicular to length. Tumors were removed and weighed after sacrifice.

### Immunohistochemistry (IHC)

Subcutaneous xenograft tumors were removed from tumor-bearing mice after sacrifice and then fixed in 4% paraformaldehyde. Tumors were then paraffin-embedded and sliced (5 mm). Sections were immunostained with appropriate primary antibody and biotin-conjugated goat anti-rabbit IgG. After the detection using DAB detection kit (Boster), slides were counterstained with hematoxylin, dehydrated and mounted. IHC staining scores were calculated as the product of the proportion of positive staining cells (0–4) and the intensity of staining (0–3). The proportion of positive staining cells was graded as followed: 0 (no staining); 1 (1–25%, including 25%); 2 (25–50%, including 50%); 3 (50–75%, including 75%); 4 (> 75%). The intensity of staining was graded as followed: negative = 0; weakly positive = 1; positive = 2; strongly positive = 3.

### Statistical analysis

Data were presented as mean ± S.E.M and all statistical analyses were carried out using GraphPad Prism 8 software. Statistical comparisons among groups in *in-vitro* experiments were evaluated by One-way or Two-way ANOVA with Post Hoc multiple comparison Tukey HSD test. For in vivo study, comparisons of tumor volumes among groups were assessed by One-way repeated measures ANOVA and Post Hoc multiple comparison Tukey HSD test. Differences of MLK3 expression levels in patients which did not follow a normal distribution was compared using the Mann–Whitney U test or Kruskal–Wallis One-way ANOVA with Dunn's multiple comparisons test. Patients’ survival was analyzed using the Kaplan–Meier method and statistical comparisons were evaluated using the Log-rank test. The distribution of categorical values within two groups was analyzed by the Chi-square test (Fisher exact test). The difference was considered to be significant at *p* < 0.05. The licenses of software are available under any requirement for permission for use.

## Results

### Elevated expression of MLK3 was found in glioblastoma cells lines and patients’ samples

**Fig. 1:**
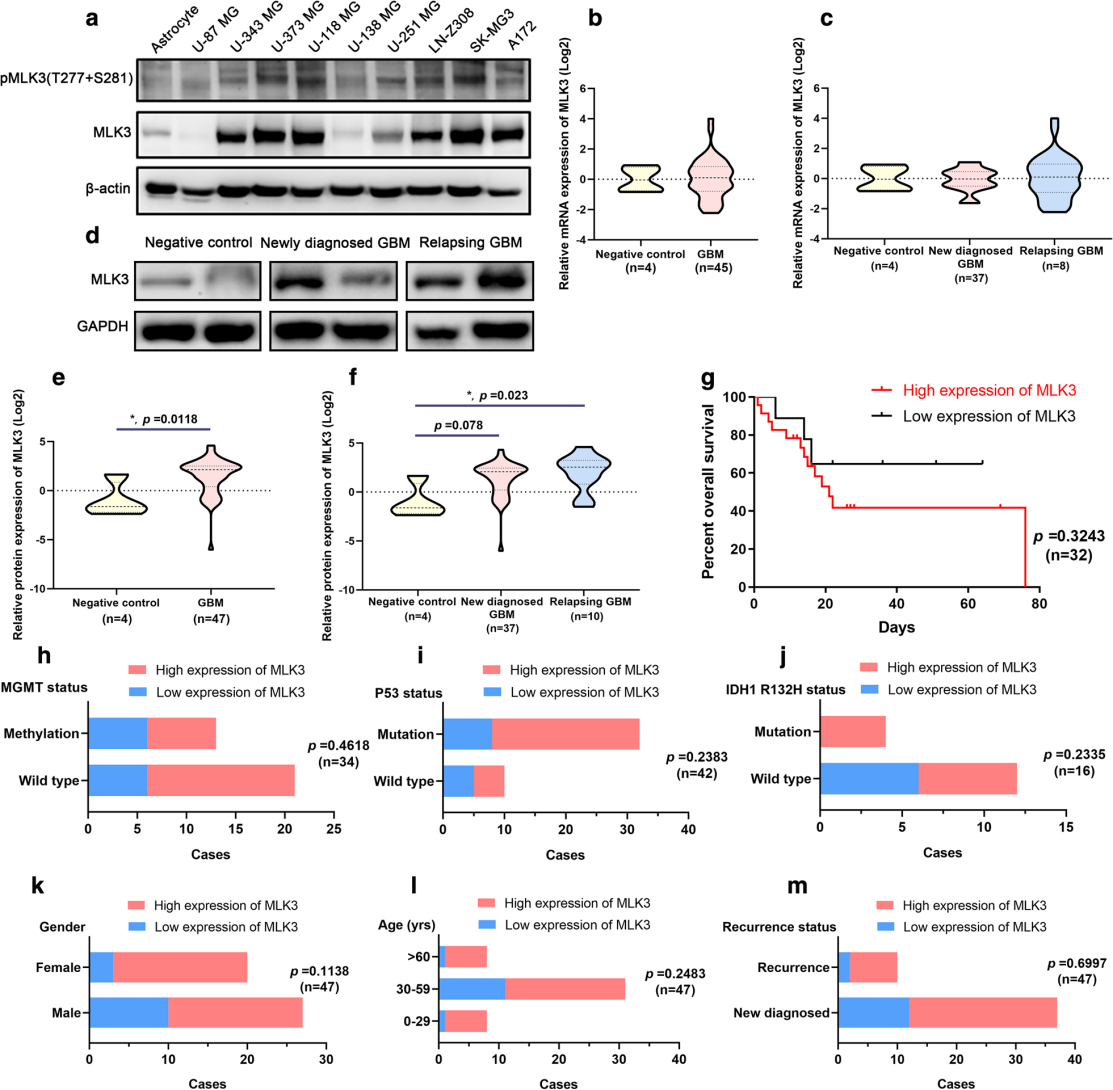
Expression pattern of MLK3 in glioblastoma and the correlation between MLK3 expression and patients’ prognosis and other clinical information. **a** Protein expression and phosphorylation of MLK3 in a set of GBM cell lines. Human astrocytes were used for comparison. Expression of β-actin was served as a loading control. **b**, **c** mRNA expression of *MLK3* gene in GBM specimens from tumor bank of the Shenzhen Second People’s Hospital. Two grade I gliomas (including 1 angiocentric glioma, 1 pilocytic astrocytoma) and two gliosis were used as the negative control. **d** Representative immunoblots of MLK3 protein expression in GBM specimens. **e**, **f** Overexpression of MLK3 was significant in 47 GBM samples, especially in 10 relapsing GBM samples. *P* values were determined by Mann–Whitney U test or Kruskal–Wallis one-way ANOVA with Dunn's multiple comparisons test. *: *p* < 0.05; (G) Kaplan–Meier survival analysis of GBM patients categorized by MLK3 expression and statistical comparisons using Log-rank test. **h**–**l** Chi-square test (Fisher exact test) was used to evaluate the correlation between MLK3 protein expression and clinical information (MGMT methylation, p53 mutation, IDH1 mutation, gender, age, and recurrence) of GBM patients

To evaluate the expression pattern of MLK3 in glioblastoma, 9 GBM cell lines and tumor tissues from 47 GBM patients were employed. Compared with normal human astrocytes, MLK3 was overexpressed in 7 of 9 GBM cell lines, except for U-87 MG and U-138 MG cells. The phosphorylation of MLK3 was elevated in U-373 MG, U-118 MG, U-251 MG, LN-Z308 and SK-MG3 cell lines (Fig. 1a). No significant difference of mRNA expression of MLK3 was found between the negative control group and GBM specimens (Fig. 1b, c). However, MLK3 protein was overexpressed in 72.34% of GBM specimens (34 of 47 cases), especially in relapsing GBM (8 of 10 cases) (fold change ≥ 2 was regarded as high expression) (Fig. 1d, f). Subsequently, we investigated the relationships between the MLK3 expression levels and the prognosis and clinical information of GBM patients. Since the contact information of some patients was missing, only 32 GBM patients completed the follow-up and were included in the Kaplan–Meier survival analysis. Results showed that the protein expression of MLK3 was not significantly correlated with overall survival of GBM patients (Fig. 1g). In addition, Chi-square test also showed that the protein expression of MLK3 was not significantly correlated with MGMT methylation, p53 mutation and IDH1 R132H status, and other clinical information (age, gender and recurrence) of GBM patients (Fig. 1h–m, Additional file 1: Table S3).

### Knockdown of MLK3 suppressed migration and invasion, but promoted adhesion of glioblastoma cells

**Fig. 2:**
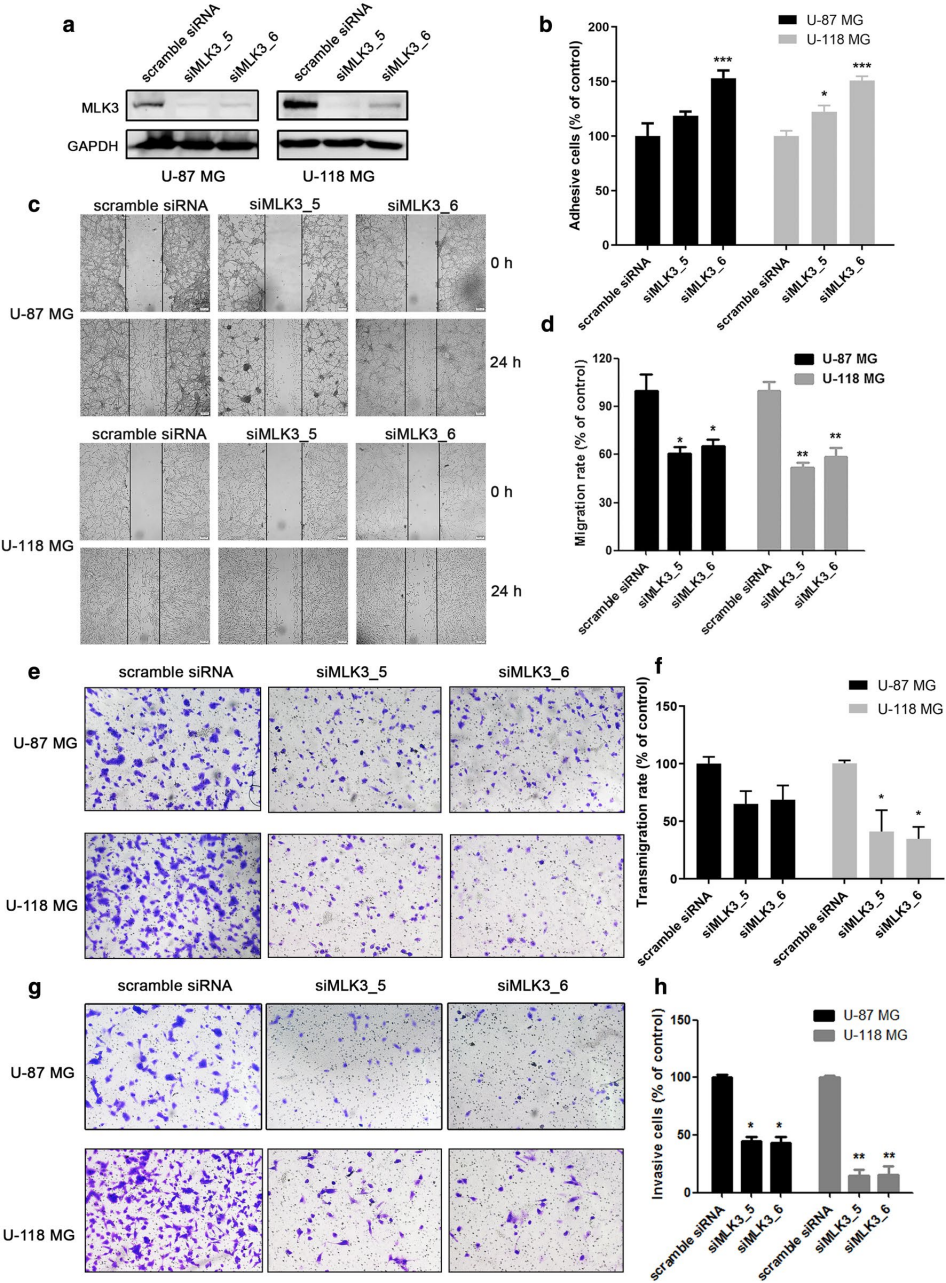
Knockdown of MLK3 markedly suppressed migration and invasion, but promoted attachment of glioblastoma cells. **a** siRNA-induced silencing of *MLK3 (MAP3K11)* gene in GBM cells U-87 MG and U-118 MG. Cells were incubated with siMLK3 duplexes (100 pmol) for 48 h. The AllStars Negative Control siRNA duplexes were denoted as “scramble siRNA” and used as a negative control. **b** Cell attachment assay showed that knockdown of MLK3 significantly increased adhesion of U-87 MG and U-118 MG cells to matrigel matrix. **c**, **d** Wound healing assay in U-87 MG and U-118 MG cells to evaluate the effect of MLK3 silencing on cell-to-cell migration. Cells were pretreated with 5 μg/mL of mitomycin C for 1 h. The lines indicate the edge of wound generated before drug treatment (0 h). Photographs were obtained at 40× magnification. **e**, **f** Boyden chamber migration assay in MLK3-silencing U-87 MG and U-118 MG cells. Representative photographs (100× magnification) showing the cells that had transmigrated through membrane to the lower surface. Cells from 5 representative fields were counted. (G-H) Boyden chamber invasion assay in MLK3-silencing U-87 MG and U-118 MG cells. Representative photographs (100× magnification) showing the invasive cells that had passed through matrigel to the lower surface of the membrane. Invaded cells from 5 representative fields were counted. n = 3; *p* values were determined by One-way ANOVA and Post Hoc multiple comparison Tukey HSD test. *: *p* < 0.05; **: *p* < 0.01; ***: *p* < 0.001

To explore the role of MLK3 in the regulation of GBM cell motility, MLK3 expression was knockdown using specific siRNA duplexes. Considering the expression and phosphorylation levels of MLK3 were increased in U-118 MG cells, but decreased in U-87 MG cells, these two cell lines with opposite MLK3 expression and phosphorylation levels were used in the following experiments. Results showed that the protein expression of MLK3 was dramatically decreased in U-87 MG and U-118 MG cells using siRNA duplexes (Fig. 2a). Wound healing and boyden chamber migration assays showed that silencing of MLK3 notably inhibited the cell-to-cell movement and transmigration of GBM cells (Fig. 2c–f). However, the attachment of U-87 MG and U-118 MG cells to the matrigel matrix was reinforced through MLK3 silencing, indicating that GBM cells may turn into quiescence due to the low expression of MLK3 (Fig. 2b). Furthermore, knockdown of MLK3 also significantly suppressed the invasive capacity of U-87 MG and U-118 MG cells (Fig. 2g, h).

### Combined inhibition of PI3Kβ and MLK3 displayed synergistic anti-proliferative effects on glioblastoma cells

**Fig. 3:**
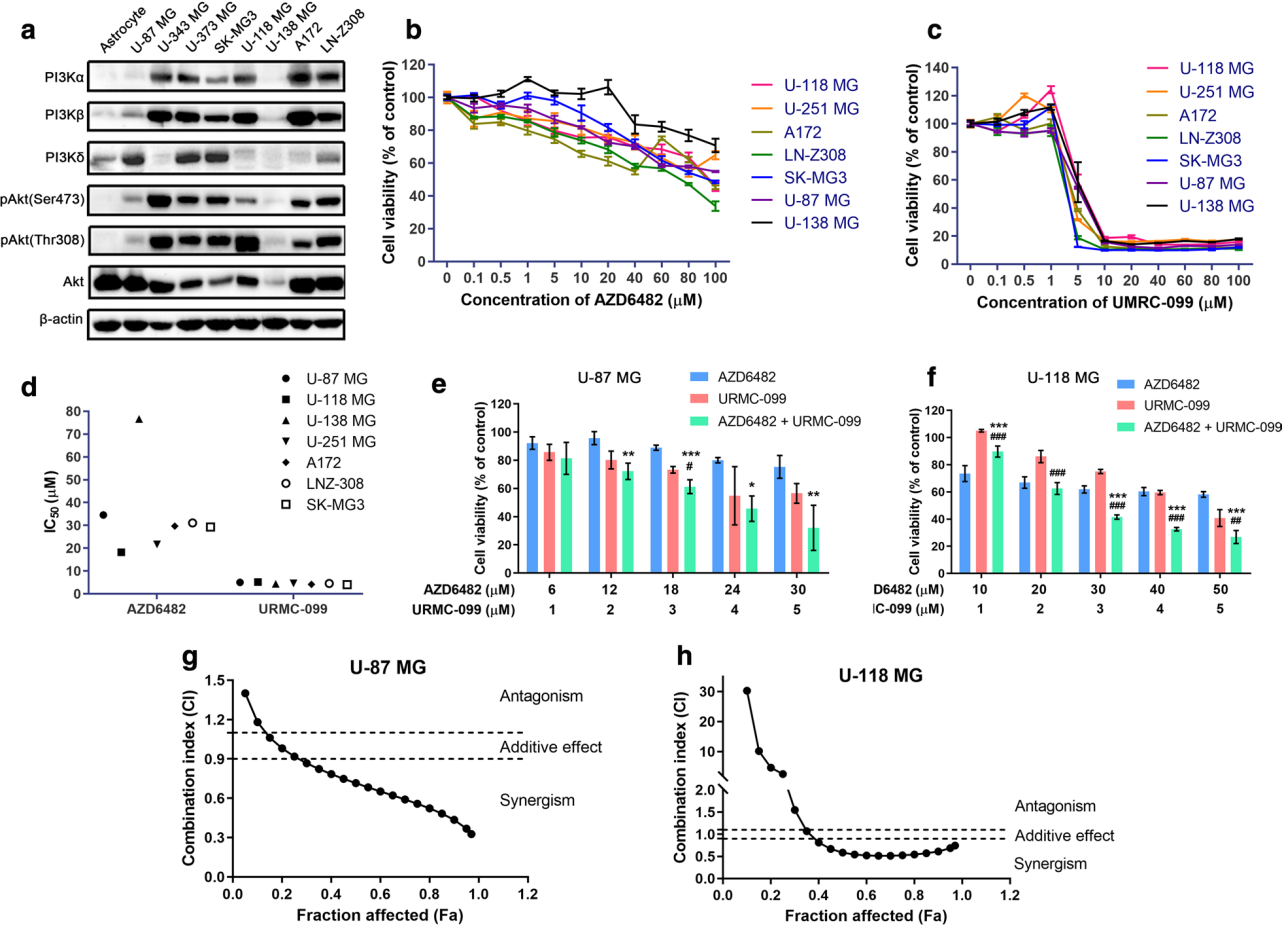
Combined inhibition of PI3Kβ and MLK3 exhibited synergistic inhibitory effect on glioblastoma cell proliferation. **a** Expression pattern of PI3K/Akt pathway molecules in a series of glioblastoma cell lines. Normal human astrocytes were used for comparison. The expression of β-actin was used as a loading control. **b**, **c** Cell proliferation assay in 7 GBM cell lines treated with AZD6482 or URMC-099 alone at different concentrations for 48 h. DMSO was used as a carrier control. **d** IC_50_ of AZD6482 and URMC-099 in 7 GBM cell lines. **e**, **f** U-87 MG and U-118 MG cells were treated with AZD6482 and URMC-099 at fixed ratios (6:1 and 10:1, respectively) for 48 h. Their combination effect on GBM cell proliferation was shown. *P* values were determined by Two-way ANOVA and Post Hoc multiple comparison Tukey HSD test. Compared with AZD6482, *: *p* < 0.05; **: *p* < 0.01; ***: *p* < 0.001; compared with URMC-099, ^#^: *p* < 0.05; ^##^: *p* < 0.01; ^###^: *p* < 0.001. **g**, **h** Fa-CI plots were automatically generated by Compusyn software according to the Chou-Talalay method, representing the CI values of combination treatment of AZD6482 and URMC-099. Two dashed lines indicated the CI values of 0.9 and 1.1

Expression and activities of key molecules in PI3K/Akt signaling pathway such as p110 isoforms and Akt were firstly evaluated in a series of GBM cell lines. It demonstrated that compared to the expression in normal human astrocytes, the expression of PI3K p110β and p110α isoforms was elevated in the most of GBM cell lines, except for U-87 MG and U-138 MG cells. Similar to PI3Kβ and PI3Kα, the phosphorylation of Akt at residues Ser473 and Thr308 was also increased in the most of GBM cell lines (Fig. 3a). Subsequently, to investigate the combination effect of PI3Kβ and MLK3 inhibitors on GBM cells, the selective PI3Kβ inhibitor AZD6482 and broad-spectrum MLK3 inhibitor URMC-099 were employed (Additional file 1: Tables S1 and S2). Generally, AZD6482 (≥ 40 μM) alone at 48-h treatment significantly inhibited the proliferation of seven GBM cell lines, in which A172 and LN-Z308 cells were more sensitive and U-138 MG cells was the most insensitive to AZD6482 (*p* ≤ 0.05)(Fig. 3b). In contrast, cell viability of seven GBM cell lines were consistently reduced after the treatment of URMC-099 (≥ 5 μM) for 48 h (*p* ≤ 0.05) (Fig. 3c). The average half maximal inhibitory concentrations (IC_50_) of AZD6482 and URMC-099 in seven GBM cell lines were 34.56 μM and 4.57 μM, respectively (Fig. 3d). Subsequently, the low PI3Kβ/MLK3 expressing (U-87 MG) and high PI3Kβ/MLK3 expressing (U-118 MG) GBM cells were treated with these two inhibitors in fixed ratios (AZD6482:URMC-099 = 6:1 and 10:1, respectively) to evaluate their combination effect on GBM cell proliferation. Results showed that either AZD6482 or URMC-099 alone markedly suppressed the proliferation of both U-87 MG and U-118 MG cells, while stronger inhibition was observed in the high PI3Kβ/MLK3 expressing U-118 MG cells. Compared with single inhibitor alone, combination of AZD6482 and URMC-099 significantly decreased the proliferation of U-87 MG and U-118 MG cells when the concentration of URMC-099 was larger than 3 μM (*p* ≤ 0.05) (Fig. 3e, f). Fa-CI plots were generated to evaluate the combination effect, revealing that combination of AZD6482 and URMC-099 displayed synergistic inhibitory effect on the proliferation of U-87 MG and U-118 MG cells (Fig. 3g, h). The minimum CI values we measured in U-87 MG and U-118 MG cells were 0.5499 and 0.4713 respectively, suggesting a strong synergism caused by concurrent inhibition of PI3Kβ and MLK3 (Tables 1 and 2). In addition, we also used the Bliss Independence Model to analyze the drug combination data. Results were in accordance with the results evaluated using CI values. The contour plots showed that the combination of AZD6482 and URMC-099 displayed synergistic effects on U-87 MG and U-118 MG cells, whereas the combination of BKM120 and URMC-099, or PQR309 and URMC-099 showed antagonism in U-251 MG and U-343 MG cells (Additional file 1: Figures S1–S3).

**Table 1:** Combination index (CI) for cell viability in GBM cell line U-87 MG treated with AZD6482 and URMC-099 (n = 3)

AZD6482 (μM)	URMC-099 (μM)	CI*
6	1	0.8339 ± 0.2241
12	2	0.9744 ± 0.1801
18	3	0.9500 ± 0.2649
24	4	0.7481 ± 0.2051
30	5	0.5499 ± 0.1539

^*^ Data were presented as mean ± S.E.M. CI < 0.9 indicates synergistic effects; CI > 1.1 indicates antagonistic effects; CI between 0.9 and 1.1 indicates additive effects

**Table 2:** Combination index (CI) for cell viability in GBM cell line U-118 MG treated with AZD6482 and URMC-099 (n = 3)

AZD6482 (μM)	URMC-099 (μM)	CI*
10	1	102.4335 ± 54.5396
20	2	1.1831 ± 0.2560
30	3	0.4915 ± 0.0643
40	4	0.4713 ± 0.0718
50	5	0.4889 ± 0.1219

^*^ Data were presented as mean ± S.E.M. CI < 0.9 indicates synergistic effects; CI > 1.1 indicates antagonistic effects; CI between 0.9 and 1.1 indicates additive effects

### The pan-PI3K inhibitor or dual PI3K/mTOR inhibitor failed to exert synergism with the MLK3 inhibitor on the proliferation of glioblastoma cells

**Fig. 4:**
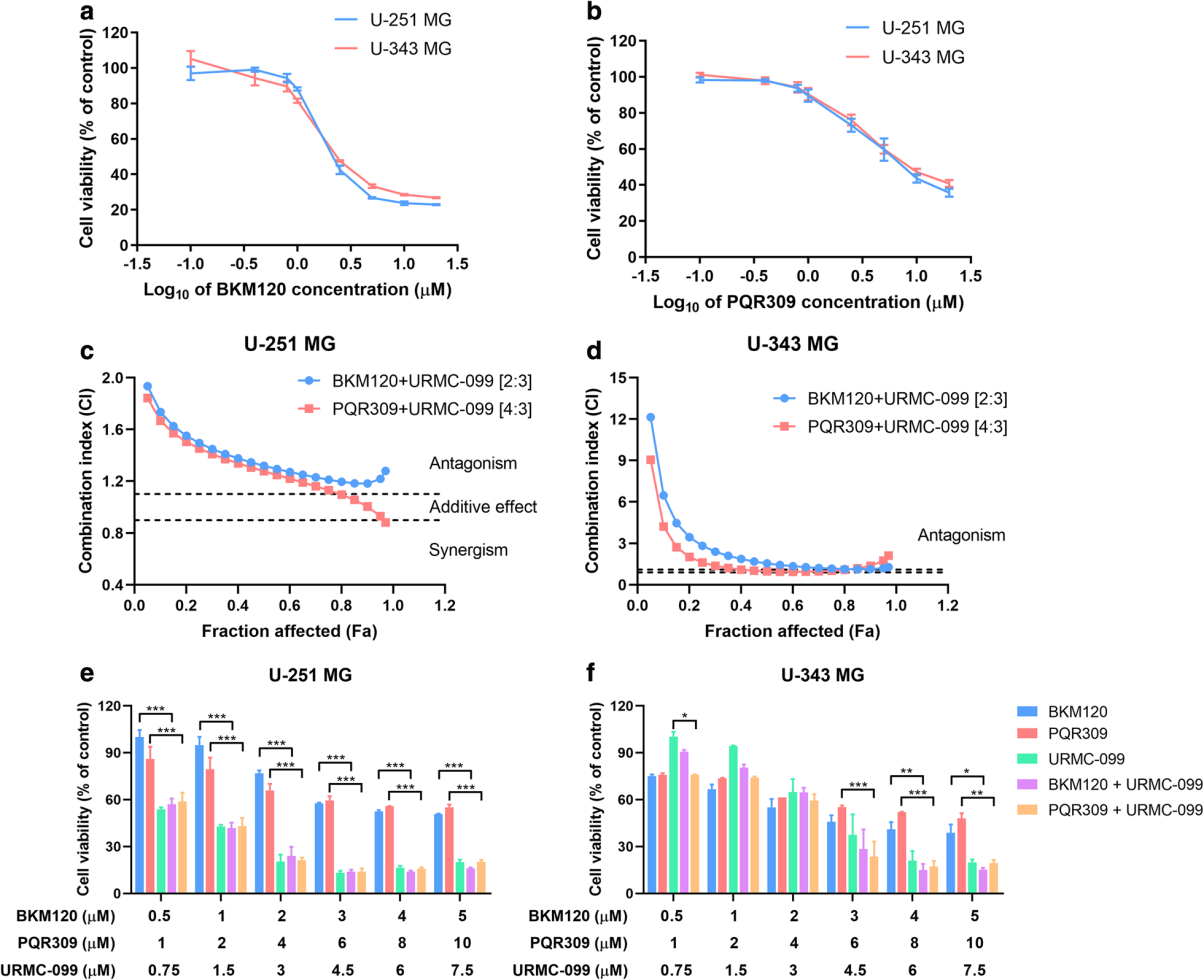
Combination of pan-PI3K and MLK3 inhibitors, or dual PI3K/mTOR and MLK3 inhibitors failed to exert synergism on glioblastoma cell proliferation. **a**, **b** U-251 MG and U-343 MG cells were treated with the pan-PI3K inhibitor BKM120 or the dual PI3K/mTOR inhibitor PQR309 alone at different concentrations. Cell viability was evaluated by MTT method in 48 h. **c**, **d** Fa-CI plots of BKM120 and URMC-099, and PQR309 and URMC-099 in fixed ratios (BKM120:URMC-099 = 2:3; PQR309:URMC-099 = 4:3). Two dashed lines indicated the CI values of 0.9 and 1.1. **e**, **f** Combination effect of BKM120 and URMC-099, or PQR309 and URMC-099 on the proliferation of U-251 MG and U-343 MG cells. Cells were treated with inhibitors for 48 h. *P* values were determined by Two-way ANOVA and Post Hoc multiple comparison Tukey HSD test. *: *p* < 0.05; **: *p* < 0.01; ***: *p* < 0.001

To investigate whether pharmacological inhibition of all class I_A_ PI3K catalytic isoforms (p110α, p110β and p110δ) or PI3K/mTOR could take the place of PI3Kβ, the pan-PI3K inhibitor BKM120 and the dual PI3K/mTOR inhibitor PQR309 were employed (Additional file 1: Table S1). Results showed that single BKM120 (≥ 1 μM) or PQR309 (≥ 2.5 μM) alone effectively suppressed the proliferation of U-251 MG and U-343 MG cells (*p* ≤ 0.05) (Fig. 4a, b). The IC_50_ values of BKM120, PQR309 and URMC-099 in both U-251 MG and U-343 MG cells were approximately 2 μM, 4 μM and 3 μM, respectively. U-251 MG and U-343 MG cells were then treated with BKM120 or PQR309 combined with URMC-099 in fixed ratios (BKM120:URMC-099 = 2:3, PQR309:URMC-099 = 4:3). We found that the CI values of BKM120 and URMC-099, or PQR309 and URMC-099 at a certain concentration were a little less than 0.9 (Table 3). However, the Fa-CI plots demonstrated that combination of BKM120 and URMC-099, or PQR309 and URMC-099 failed to exhibit synergistic anti-prolierative effect on U-251 MG and U-343 MG cells (Fig. 4c, d). In addition, compared with single inhibitor alone, combination of BKM120 and URMC-099, or PQR309 and URMC-099 didn’t significantly decrease cell proliferation of U-251 MG and U-343 MG cells (Fig. 4e, f).

**Table 3:** Combination index (CI) for cell viability in GBM cell lines U-251 MG and U-343 MG treated with BKM120/PQR309 and URMC-099 (n = 3)

BKM120 (μM)	PQR309 (μM)	URMC-099 (μM)	CI* in U-251 MG cells		CI in U-343 MG cells	
			BKM120 + URMC-099	PQR309 + URMC-099	BKM120 + URMC-099	PQR309 + URMC-099
0.5	1	0.75	1.3989 ± 0.2163	1.5254 ± 0.3801	4.7114 ± 0.2524	1.1346 ± 0.0512
1	2	1.5	1.4334 ± 0.1484	1.4664 ± 0.2958	3.6418 ± 0.8218	1.9790 ± 0.0174
2	4	3	1.1565 ± 0.2646	0.8072 ± 0.0409	2.9486 ± 0.6378	1.7963 ± 0.2530
3	6	4.5	0.8357 ± 0.0579	0.6518 ± 0.0092	1.2952 ± 0.5308	0.8334 ± 0.1518
4	8	6	1.1241 ± 0.1438	1.0504 ± 0.1408	0.9371 ± 0.0928	0.8804 ± 0.0166
5	10	7.5	1.6589 ± 0.2057	1.8739 ± 0.1506	1.1621 ± 0.0186	1.1876 ± 0.0811

^*^ Data were presented as mean ± S.E.M. CI < 0.9 indicates synergistic effects; CI > 1.1 indicates antagonistic effects; CI between 0.9 and 1.1 indicates additive effects

### Combination of PI3Kβ and MLK3 inhibitors suppressed migration/invasion of GBM cells and impeded the formation of lamellipodia and focal adhesion

**Fig. 5:**
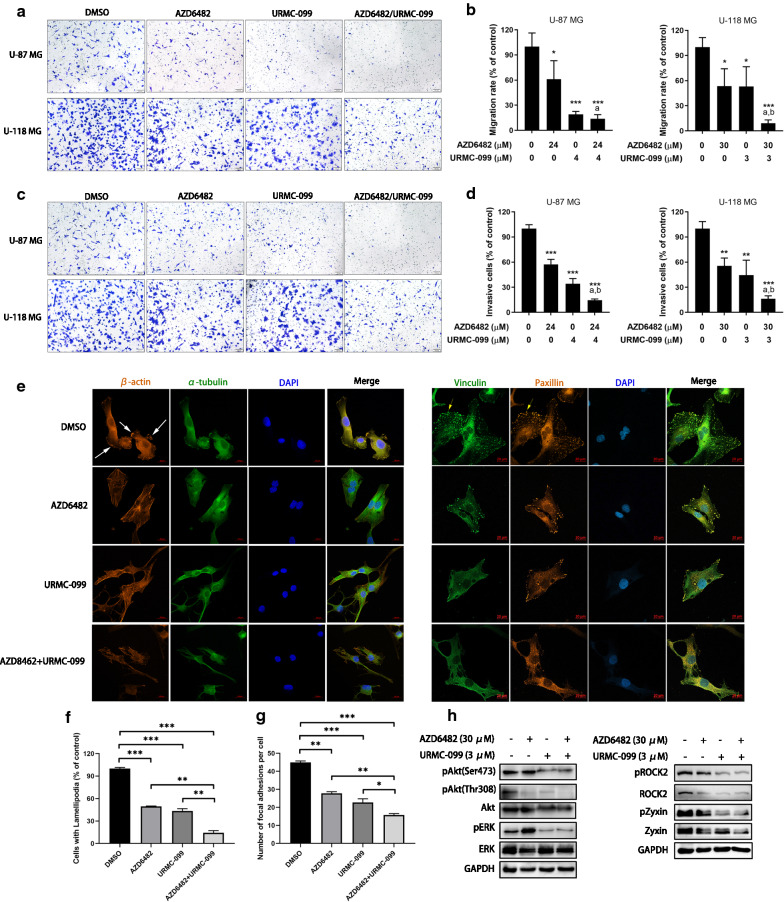
Combined inhibition of PI3Kβ and MLK3 suppressed cell migration and invasion, and blocked the formation of lamellipodia and focal adhesions. **a**, **b** Boyden chamber migration assay in U-87 MG and U-118 MG cells treated with AZD6482 and URMC-099 for 6 h. **c**, **d** Boyden chamber invasion assay in U-87 MG and U-118 MG cells treated with AZD6482 and URMC-099 for 24 h. *P* values were determined by One-way ANOVA and Post Hoc multiple comparison Tukey HSD test. Compared with DMSO, *: *p* < 0.05; ***: *p* < 0.001; compared with AZD6482, ^a^: *p* < 0.05; compared with URMC-099, ^b^: *p* < 0.05. **e** Immunofluoresescence of U-118 MG cells after treatment with AZD6482 (30 μM) and URMC-099 (3 μM) for 3 h. Representative photographs show the effects of AZD6482 and URMC-099 on the formation of lamellipodia protrusions (white arrows) and FAs (yellow arrows). Bar = 20 μm. **f** Number of U-118 MG cells with lamellipodia was decreased by the combination of the combination of AZD6482 and URMC-099. **g** Number of FAs per U-118 MG cell was reduced by the combination of the combination of AZD6482 and URMC-099. *P* values were determined by One-way ANOVA and Post Hoc multiple comparison Tukey HSD test. *: *p* < 0.05; **: *p* < 0.01; ***: *p* < 0.001. (H) Immunoblotting of U-118 MG cells treated with AZD6482 (30 μM) and URMC-099 (3 μM) alone or in combination for 24 h. Expression of GAPDH was served as a loading control. Data were representative of two independent experiments

To investigate whether the combined inhibition of PI3Kβ and MLK3 could synergistically inhibit GBM cell migration and invasion, U-87 MG cells were treated with 24 μM of AZD6482 and 4 μM of URMC-099, while U-118 MG cells were treated with 30 μM of AZD6482 and 3 μM of URMC-099, since cells treated with inhibitors at these concentrations showed synergistic effect but less cytotoxicity. Results of boyden chamber migration and invasion assays showed that AZD6482 or URMC-099 alone notaly decreased migration and invasion of U-87 MG and U-118 MG cells. Compared with the single inhibitor, combination of AZD6482 or URMC-099 significantly potentiated the inhibition of GBM cell migration and invasion (*p* ≤ 0.05) (Fig. 5a–d). Lamellipodia and focal adhesions are essential to cell migration. To study whether the combination of PI3Kβ and MLK3 inhibitors could affect the formation of lamellipodia and focal adhesions, U-118 MG cells were treated with AZD6482 and URMC-099 for 3 h. Lamellipodia were labelled with actin filaments staining, while focal adhesions were visualized by vinculin and paxillin staining. It was found that AZD6482 or URMC-099 alone reduced the number and size of lamellipodia and focal adhesions in U-118 MG cells, and their combination significantly reinforced this effect (Fig. 5e–g). In addition, U-118 MG cells treated with the combination of AZD6482 and URMC-099 displayed shrunken morphology and disorganized actin bundles (Fig. 5e). We also found that either AZD6482 or URMC-099 decreased the phosphorylation of Akt at residues Ser473 and Thr308, as well as the protein expression of ROCK2 and Zyxin. Interestingly, URMC-099 reduced but AZD6482 conversely increased the phosphorylation of ERK at residues Thr277 and Ser281, indicating that blockade of PI3K signaling may give a positive feedback to activate MAPK signaling. Similar to URMC-099, combination of AZD6482 and URMC-099 also decreased the phosphorylation of Akt and ERK, as well as the protein expression of ROCK2 and Zyxin (Fig. 5h).

### Tumor growth of glioblastoma xenograft was effectively reduced by the combination of PI3Kβ and MLK3 inhibitors

**Fig. 6:**
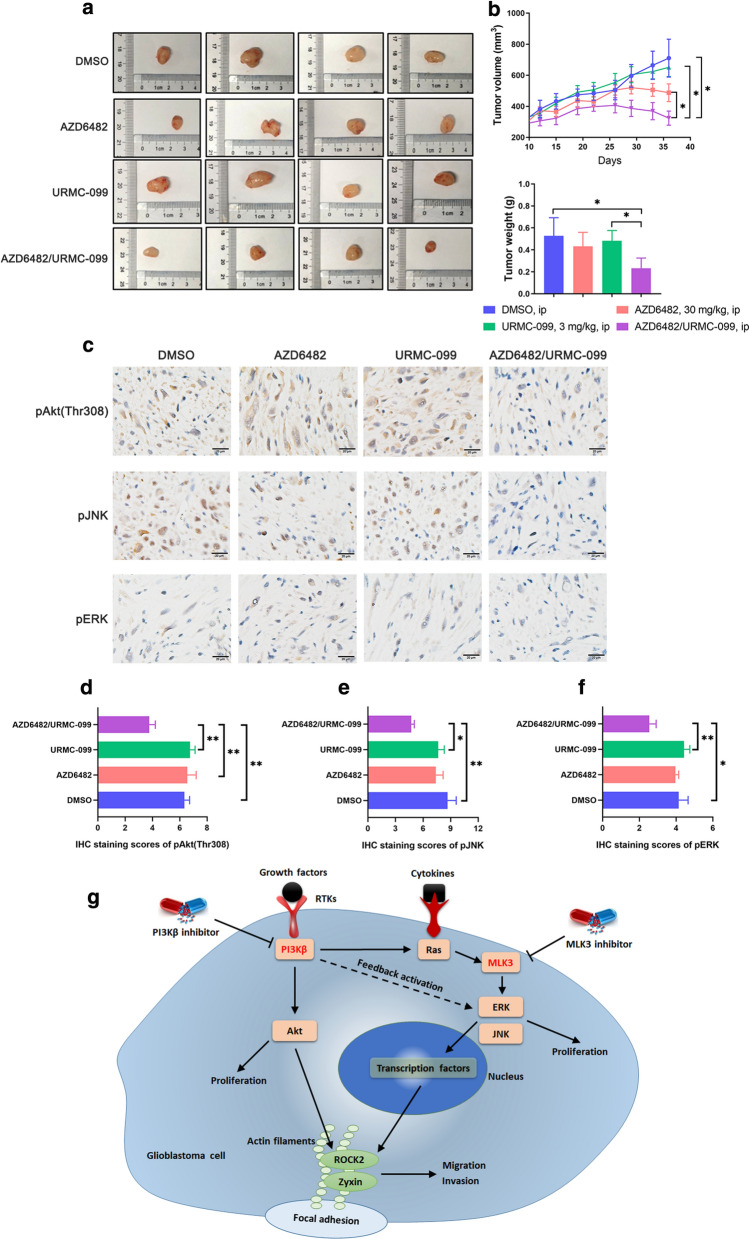
Combined inhibition of PI3Kβ and MLK3 effectively suppress glioblastoma xenograft growth in vivo. **a** Glioblastoma cells U-118 MG (5 × 10^6^ cells) were subcutaneously injected into the flank of Balb/C nude mice. Mice were intraperitoneally (i.p.) injected once daily for 7 days with vehicle, AZD6482 (30 mg/kg) and URMC-099 (3 mg/kg) alone or in combination. Measurement of tumor volumes started on the day of the first administration. **a** Representative photographs show the size of subcutaneous tumor xenografts from mice sacrificed after 36-day post-administration. **b** Tumor volumes from 12-day post-administration to the end of the experiment, and tumor weight of tumor xenografts after sacrifice in 36-day post-administration were measured. n = 6; statistical difference of tumor volumes was determined by One-way repeated measures ANOVA and Post Hoc multiple comparison Tukey HSD test, while difference of tumor weight was determined by One-way ANOVA and Post Hoc multiple comparison Tukey HSD test. *: *p* < 0.05. **c** Immunohistochemistry analysis of the phosphorylation of Akt, JNK and ERK in representative sections of tumor xenografts after sacrifice. Bar = 20 μm. **d**–**f** IHC staining scores of the phosphorylation of Akt, JNK and ERK in tumor sections. *P* values were determined by One-way ANOVA and Post Hoc multiple comparison Tukey HSD test. *: *p* < 0.05; **: *p* < 0.01. **g** Simplified schematic representation demonstrating a crosstalk between PI3Kβ and MLK3. Concurrent inhibition of these two molecules displayed synergistic anti-glioblastoma effects through inhibition of Akt, ERK, ROCK2 and Zyxin

To evaluate the combination effect of PI3Kβ and MLK3 inhibitors in vivo, Balb/C nude mice bearing subcutaneous U-118 MG glioblastoma xenograft were intraperitoneally injected with vehicle, AZD6482 (30 mg/kg), URMC-099 (3 mg/kg), and the combination of AZD6482 (30 mg/kg) and URMC-099 (3 mg/kg), respectively. No severe adverse reaction was observed in mice with drug treatment until sacrifice. Neither AZD6482 nor URMC-099 alone significantly suppressed U-118 MG xenograft tumor growth. However, compared with single inhibitor alone, combination of AZD6482 and URMC-099 effectively decreased tumor volume after 26-day post-administration (*p* < 0.05). Tumor weight and size were also reduced by the combination of AZD6482 and URMC-099 after sacrifice in 36-day post-administration (*p* < 0.05) (Fig. 6a, b). Immunohistochemistry analysis of xenograft showed that the phosphorylation levels of Akt at Thr308, JNK and ERK have no obvious changes after the treatment of AZD6482 or URMC-099 alone, but significantly decreased by their combination treatment (Fig. 6c–f). However, there is no significant difference of the phosphorylation of Akt at Ser473 and pROCK2 at Ser1366 between combination treatment and single inhibitor alone (Additional file 1: Figure S4). Taken together, this study was summarized in a schematic diagram, showing that PI3Kβ has a crosstalk with MLK3. PI3Kβ could activate MAPK signaling through Ras, while inhibition of PI3Kβ could give a positive feedback to activated ERK. Concurrent pharmacological inhibition of PI3Kβ and MLK3 blocks the activation of Akt and ERK/JNK, leading to the impaired GBM cell proliferation. On the other side, activated of ERK and JNK could translocate into the nucleus and interact with the transcription factors, which may then increase the expression of Zyxin and ROCK2. In addition, PI3Kβ/Akt signaling could mediate the activation of Zyxin and ROCK2. Thus, inhibition of MLK3 and PI3Kβ may prevent these processes, leading the blockade of GBM cell migration and invasion (Fig. 6g).

## Discussion

Extensive infiltration and abnormal invasive capacity of glioblastoma cells are the main causes of tumor recurrence. MLK3 plays essential roles in tumorigenesis, migration, invasion and metastasis of cancer cells. Studies found that MLK3 enhanced the phosphorylation of the focal adhesion protein paxillin on Ser 178 and Tyr118, and inhibition of MLK3/JNK/paxillin signaling axis decreased Rho activity to block focal adhesion turnover and migration of breast cancer cells [15]. Inhibition of MLK3 downregulated the expression of FRA-1, MMP-1 and MMP-9, leading to the transendothelial migration and matrigel invasion of triple-negative breast cancer cells [23]. However, there are few studies focused on the roles of MLK3 in glioblastoma currently. In this study, we found that the protein expression of MLK3 was elevated in most of GBM patients, especially in patients with GBM recurrence, whereas the mRNA expression of MLK3 was not. It indicates that post-transcriptional modification and the regulation of MLK3 in translation level may determine its high protein expression in GBM. However, no significance between MLK3 expression and overall survival or other clinical information was showed, which may be caused by the small sample size of GBM patients. And then we found that knockdown of MLK3 expression could inhibited migration and invasion, and promoted adhesion of GBM cells. Misek et al. also found that pharmacological inhibition or silencing of MLK3 impeded EGF-induced migration and invasion, and deceased JNK activity in GBM cells, which is inconsistent with our findings [24].

The crosstalk between PI3K/Akt and MAPK signaling pathways is complicated. Combination strategies using PI3K and MAPK inhibitors are widely investigated for cancer treatment. Our previous study found that combination of PI3Kβ and JNK inhibitors displayed synergistic inhibitory effects on GBM cell proliferation and migration [10]. Besides, combined inhibition of PI3K and MEK/ERK also shows synergism and have entered into clinical trials in patients with solid tumors. In 11 phase I clinical trials of 236 patients with advanced solid tumors, combination of PI3K/Akt and MEK/ERK pathways inhibitors produced better clinical benefits to patients with *PTEN* deletions and *KRAS*/*BRAF* mutations [20]. In a phase Ib clinical trial of patients with selected advanced solid tumors, combination of the pan-PI3K inhibitor BKM120 and the MEK1/2 inhibitor trametinib displayed promising anti-tumor activities to patients with *KRAS*-mutant ovarian cancer [25]. In addition, concurrent inhibition of PI3Kβ and MEK significantly reduced tumor growth and prolonged the survival of *Pten;Trp53*-null mice bearing sarcomatoid malignant mesothelioma [26]. These findings suggest that combined inhibition of PI3K/Akt and MAPK signaling pathways has synergistic anti-tumor effects and may be a promising therapeutic approach for cancer treatment. Here, this study demonstrated that combination of the PI3Kβ inhibitor AZD6482 and the MLK3 inhibitor URMC-099 showed synergistic inhibitory effects on the proliferation, migration and invasion of GBM cells U-87 MG and U-118 MG. AZD6482 is a selective PI3K p110β inhibitor that is used as an antiplatelet agent for prophylaxis of thrombotic disorders in phase I clinical trial [27]. Similar to another selective PI3Kβ inhibitor TGX-221, we found that AZD6482 moderately inhibited cell proliferation of multiple GBM cell lines, which is owing to the kinase-independent function of PI3Kβ isoform [10, 28]. Due to the lack of specific MLK3 inhibitor, a broad-spectrum MLK inhibitor URMC-099 was used in this study, which is a blood–brain barrier (BBB) penetrant targeting MLK1, MLK2, MLK3 and DLK [29]. Our previous study found that the CI value of PI3Kβ inhibitor TGX-221 (20 μM) and JNK inhibitor SP600125 (20 μM) in U-87 MG cells was 0.855 [10]. In this study, the minimum CI value of AZD6482 and URMC-099 in U-87 MG and U-118 MG cells was 0.5499 and 0.4713 respectively, indicating a stronger synergism compared with TGX-221 and SP600125.

Focal adhesion (FA) is a cytoskeletal structure consisting of numerous proteins that provide a strong linkage between actin cytoskeleton and the extracellular matrix (ECM). FA scaffold proteins, such as paxillin, talin, zyxin, α-actinin and vinculin, participate in the formation and turnover of FAs, which generate the tension and traction force to alter cell morphology and move the cell body forward [30]. In the stage of FA formation, talin binds to integrin and F-actin, and then recruits vinculin and α-actinin to stabilize and reinforce the connection of cytoskeleton and ECM [31, 32]. In this study, we stained vinculin and paxillin to visualize FAs and found that combination of AZD6482 and URMC-099 significantly decreased the number and size of FAs in GBM cells, compared with the single inhibitor alone. It suggested that formation and maturation of FAs were impeded by this drug combination, leading to the transformation of GBM cells from active to quiescent status. Furthermore, we showed that combination of AZD6482 and URMC-099 sharply reduced the protein expression and phosphorylation of Zyxin and ROCK2. Zyxin is a focal adhesion protein that maintains FAs and actin stress fibers through interacting with Enabled/vasodilator-stimulated phospho-protein (Ena/VASP) [33]. Knockdown of Zyxin in chondrocytes inhibited actin polymerization and FA maintenance by promoting the transformation of F-actin to G-actin and decreasing the expression of vinculin [34]. ROCK2, a member of ROCK family and the downstream effector of RhoA, is involved in the regulation of various cancer cell contraction, migration, invasion and survival [35]. Inhibition of ROCK2 suppressed myoblast migration through impeding FA maturation and the recruitment of ROCK2 to FA sites [36]. In addition, Src-mediated phosphorylation of ROCK2 at tyrosine 722 was essential to FA turnover and cell contraction of NIH3T3 cells [37]. Thus, combination of AZD6482 and URMC-099 may prevent focal adhesions from formation and maturation via reducing the expression of Zyxin and ROCK2 in GBM cells.

Compared with the single inhibitor alone, combination of AZD6482 and URMC-099 also significantly inhibited the tumor growth in mice bearing GBM xenografts. Since AZD6482 and other PI3Kβ inhibitors are not able to penetrate the BBB, the application of this combination strategy in the intracranial GBM xenograft is limited. In order to solve this problem, we used the BBB-penetrating pan-PI3K inhibitor BKM120 and dual PI3K/mTOR inhibitor PQR309 to investigate whether they can replace AZD6482, but the combination of URMC-099 and BKM120, or URMC-099 and PQR309 failed to show synergism. Similarly, our previous study found that combined inhibition of PI3Kβ isoform and JNK showed synergistic inhibitory effect, whereas the combination of PI3Kα isoform and JNK inhibitors displayed antagonism [10]. Considering that both BKM120 and PQR309 inhibit all PI3K isoforms (p110α, p110β and p110δ) and p110α occupies a dominant place, the failure of the combination between URMC-099 and BKM120 or PQR309 may be resulted from the antagonistic crosstalk between PI3Kα and MLK3/JNK.

## Conclusion

In summary, MLK3 was overexpressed in newly diagnosed and relapsing GBM specimens. Silencing of MLK3 notably inhibited the migration and invasion, but reinforced the adhesion of GBM cells. Concurrent pharmacological inhibition of PI3Kβ and MLK3 using AZD6482 and URMC-099 exhibited synergistic inhibitory effects on *in-vitro* GBM cell proliferation, migration and invasion, as well as *in-vivo* xenograft growth. This combination strategy also markedly impeded the formation of lamellipodia and FAs, and decreased the phosphorylation of Akt and ERK as well as the protein expression of ROCK2 and Zyxin. Taken together, combined inhibition of PI3Kβ and MLK3 may be a promising therapeutic approach for GBM treatment, but an effective and BBB-penetrating drug delivery system is required prior to the clinical application.

## Supplementary Information

**Additional file 1.** Additional tables and figures.

## Data Availability

Not applicable.

## Abbreviations

α-MEM: Minimum essential medium alpha
BBB: Blood–brain barrier
BSA: Bovine serum albumin
CI: Combination index
DLK: Dual leucine zipper-bearing kinase
DMSO: Dimethyl sulfoxide
ECM: Extracellular matrix
Ena/VASP: Enabled/vasodilator-stimulated phospho-protein
ERK: Extracellular signal-regulated kinase
Fa: Fraction affected
FA: Focal adhesion
FBS: Fetal bovine serum
GBM: Glioblastoma multiforme
IC_50_: Half maximal inhibitory concentration
JNK: C-Jun N-terminal kinase
MAPK: Mitogen-activated protein kinase
MAP3K: Mitogen-activated protein kinase kinase kinase
MLK: Mixed lineage kinase
MTT: 3-(4,5-Dimethylthiazol-2-yl)-2,5-diphenyltetrazolium bromide
PBS: Phosphate-buffered saline
PI3K: Phosphatidylinositol 3-kinase
PI3Kβ: PI3K p110β isoform
qRT-PCR: Quantitative real-time PCR
ROCK2: Rho-associated protein kinase 2
siRNA: Small interfering RNA
WHO: World Health Organization
ZAK: Zipper sterile-α motif kinase
