# Correction to: Synergism between the phosphatidylinositol 3-kinase p110β isoform inhibitor AZD6482 and the mixed lineage kinase 3 inhibitor URMC-099 on the blockade of glioblastoma cell motility and focal adhesion formation

**DOI:** 10.1186/s12935-021-02028-1

**Published:** 2021-07-28

**Authors:** Hua‑fu Zhao, Chang‑peng Wu, Xiu‑ming Zhou, Peng‑yu Diao, Yan‑wen Xu, Jing Liu, Jing Wang, Xian‑jian Huang, Wen‑lan Liu, Zhong‑ping Chen, Guo‑dong Huang, Wei‑ping Li

**Affiliations:** 1 Department of Neurosurgery, Shenzhen Second People’s Hospital/The First Affiliated Hospital of Shenzhen University Health Science Center, Shenzhen, 518035 China; 2 Department of Neurosurgery, People’s Hospital of Longhua District, Shenzhen, 518109 China; 3 Epilepsy Center, Guangdong 999 Brain Hospital, Guangzhou, 510510 China; 4 Department of Neurosurgery/Neuro‑Oncology, Sun Yat-Sen University Cancer Center, State Key Laboratory of Oncology in South China, Collaborative Innovation Center for Cancer Medicine, Guangzhou, 510060 China

## Correction to: Cancer Cell Int (2021) 21:24 10.1186/s12935-020-01728-4

Following the publication of the original article [1], we were notified of a mistake in Fig. 5c.

Figure 5c contains the representative images of cell invasion, while Fig. 5a contains the representative images of cell migration. Figure 5a and 5c were both taken at the high magnification (10×). By mistake, the images in the cell migration folder were used in the Fig. 5c. Therefore, Fig. 5a and the statistical histograms in Fig. 5b and 5d are correct, but all the images in Fig. 5c were incorrect.

The conclusions made are not influenced by the mistake of Fig. 5c, because the statistical analysis was performed based on the images of cell migration and invasion taken at the low magnification (4×).

The original article has been corrected to reflect these changes.
