# Supplementary material for: Synergism between the phosphatidylinositol 3-kinase p110β isoform inhibitor AZD6482 and the mixed lineage kinase 3 inhibitor URMC-099 on the blockade of glioblastoma cell motility and focal adhesion formation
Source: Cancer Cell Int. 2021 Jan 6;21:24. doi: 10.1186/s12935-020-01728-4 (PMC7789614; doi:10.1186/s12935-020-01728-4)
Supplement: Supplementary file 1 — Additional file 1. Additional tables and figures. [file 12935_2020_1728_MOESM1_ESM.docx]

**Supplementary Table 1. Characteristics of PI3K inhibitors AZD6482, BKM120 and PQR309.**

| **Compound** | **Structure** | **IC_50_ (nM)** | | | | | **Reference** |
| --- | --- | --- | --- | --- | --- | --- | --- |
|  |  | **p110α** | **p110β** | **p110δ** | **p110γ** | **mTOR** |  |
| AZD6482 | 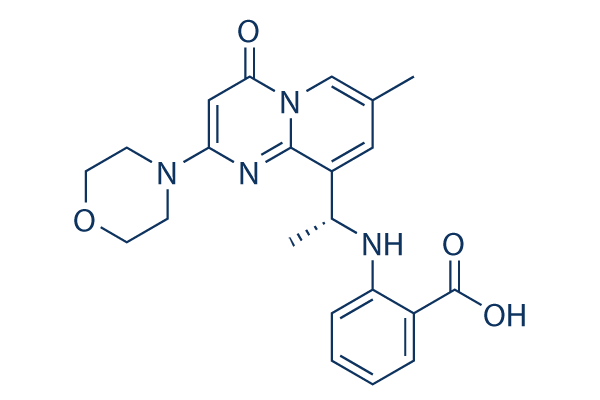 | 870 | 10 | 80 | 1090 | N/A | [5,3] |
| BKM120 | 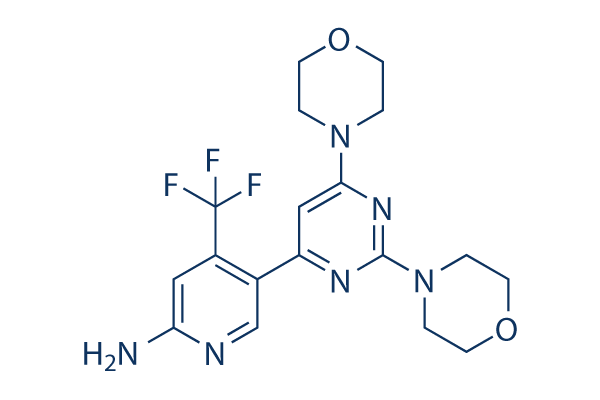 | 52 | 166 | 116 | 262 | 4600 | [2] |
| PQR309 | 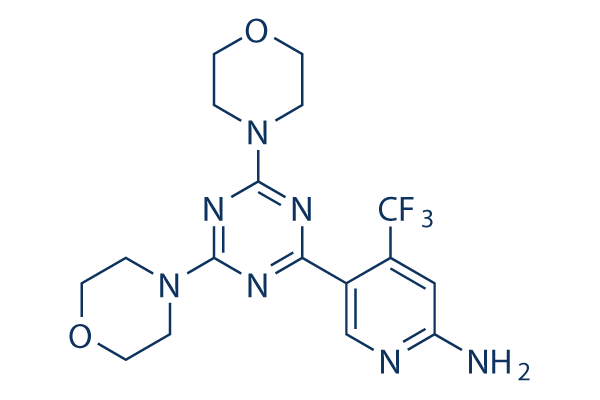 | 1.5 | 11 | 25 | 25 | 12 | [1] |

N/A, not available.

**Supplementary Table 2. Characteristics of MLK3 inhibitor URMC-099.**

| **Compound** | **Structure** | **IC_50_ (nM)** | | | | | | **Reference** |
| --- | --- | --- | --- | --- | --- | --- | --- | --- |
|  |  | **Abl1** | **LRRK2** | **MLK3** | **MLK1** | **VEGFR1/FLT1** | **MLK2** |  |
| URMC-099 | 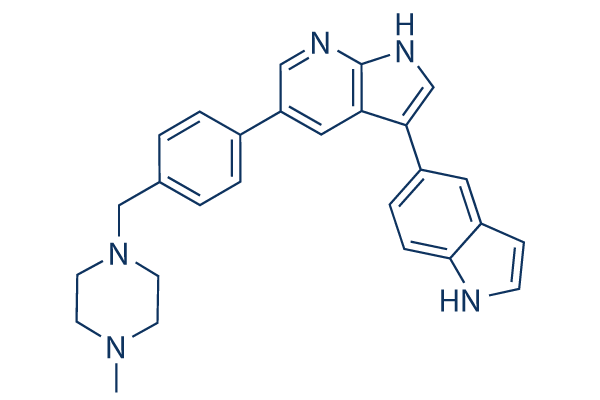 | 6.5 | 11 | 14 | 19 | 39 | 42 | [4] |

**Supplementary Table 3. Clinical information of patients with glioblastoma multiforme and their correlation with MLK3 expression.**

|  |  |  | **No. of cases** | ***P* value** |
| --- | --- | --- | --- | --- |
| Gender | Male |  | 27 | 0.1138 |
|  | Female |  | 20 |  |
| Age | 0-29 |  | 8 | 0.2483 |
|  | 30-59 |  | 31 |  |
|  | >60 |  | 8 |  |
| Recurrence | Yes |  | 10 | 0.6997 |
|  | No |  | 37 |  |
| MGMT status | Wild type |  | 21 | 0.4618 |
|  | Mutation |  | 13 |  |
| P53 status | Wild type |  | 10 | 0.2383 |
|  | Mutation |  | 32 |  |
| IDH1/2 status | Wild type |  | 12 | 0.2335 |
|  | Mutation |  | 4 |  |
| Total |  |  | 47 |  |


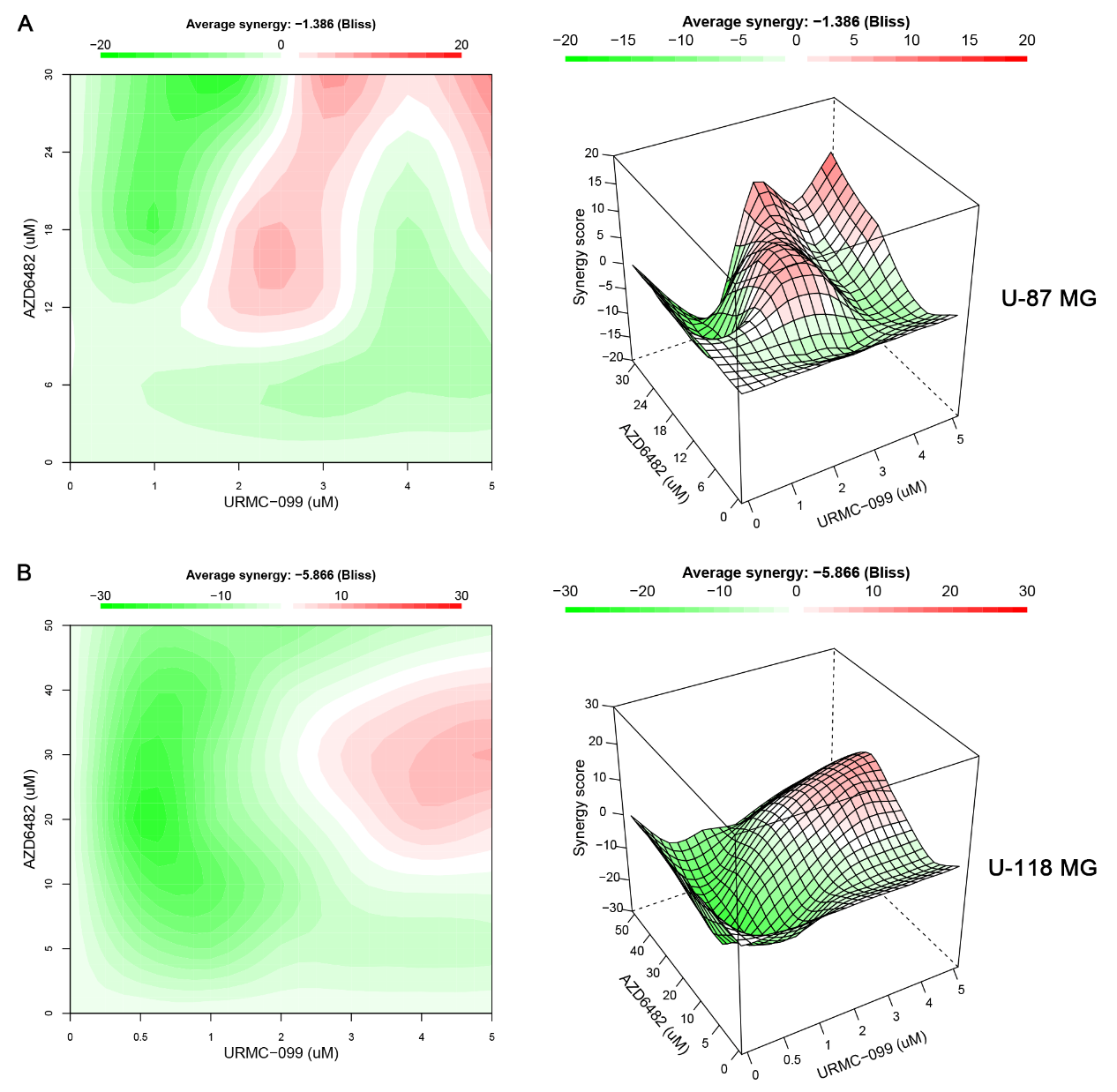


**Supplementary Figure 1: Contour plot of Bliss model synergy index in for AZD6482 and URMC-099 combination in U-87 MG (A) and U-118 MG (B) cells.** Red indicates synergism, while green represents antagonism.


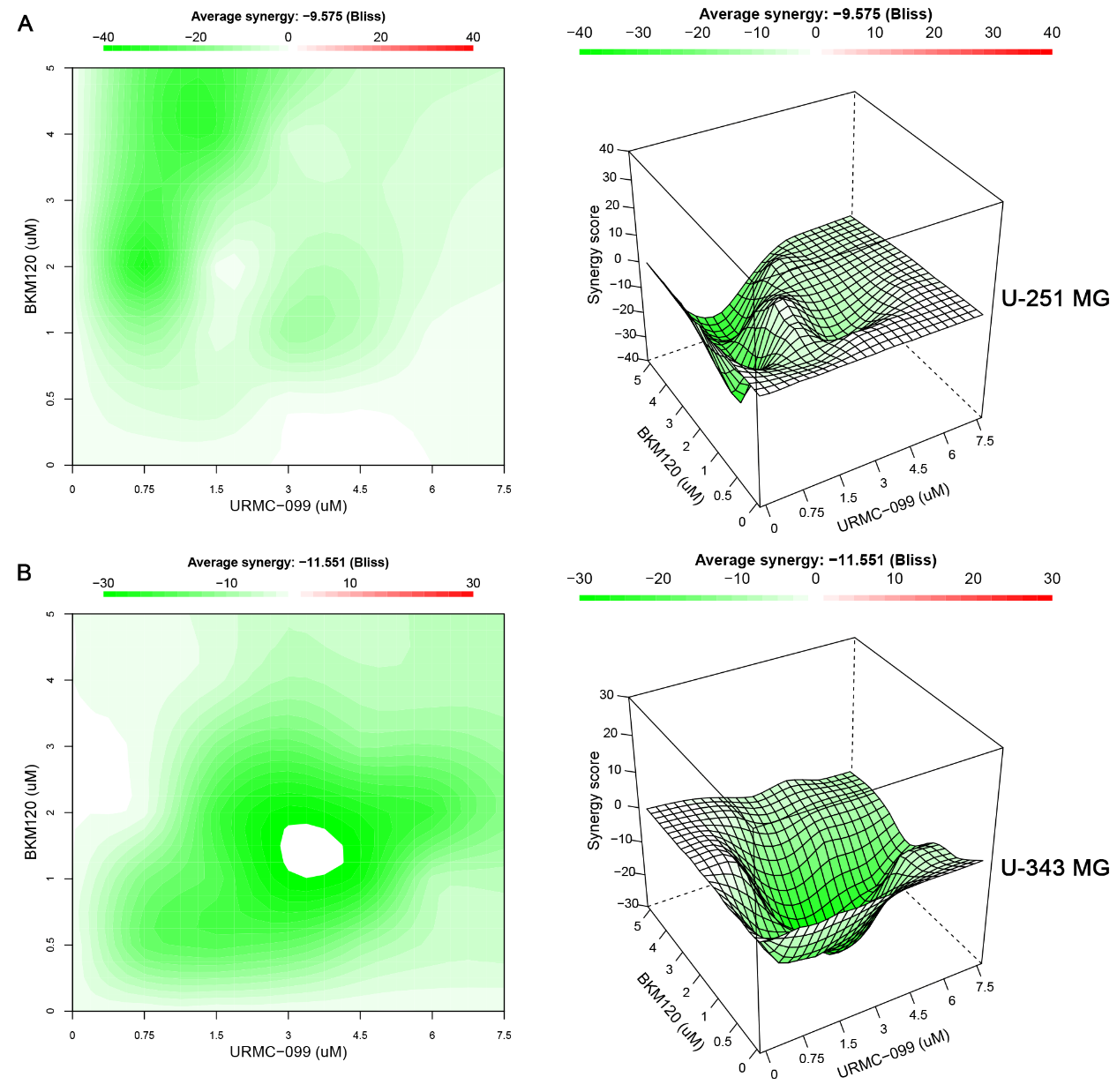


**Supplementary Figure 2: Contour plot of Bliss model synergy index in for BKM120 and URMC-099 combination in U-251 MG (A) and U-343 MG (B) cells**. Red indicates synergism, while green represents antagonism.


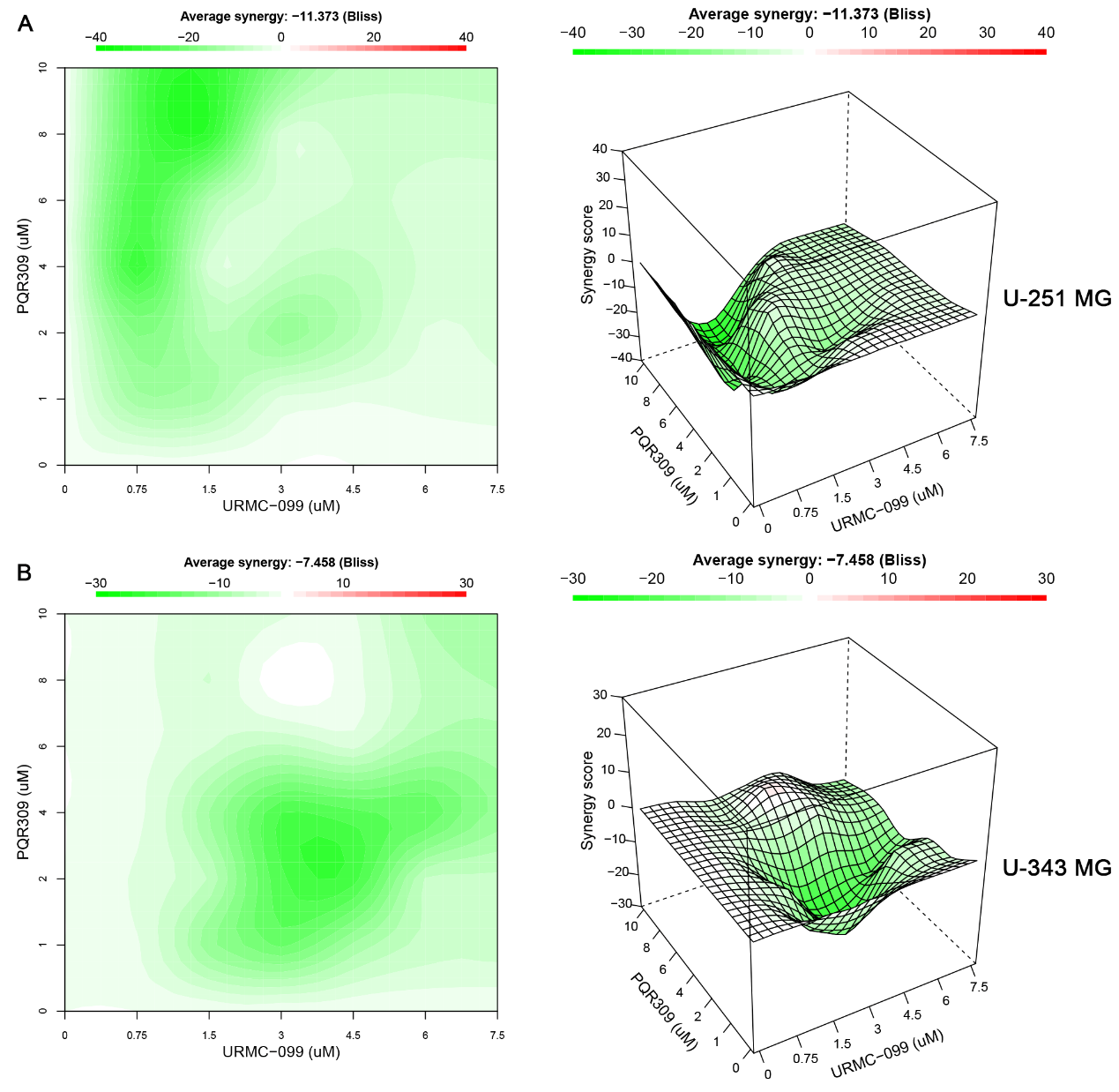


**Supplementary Figure 3: Contour plot of Bliss model synergy index in for PQR-309 and URMC-099 combination in U-251 MG (A) and U-343 MG (B) cells.** Red indicates synergism, while green represents antagonism.


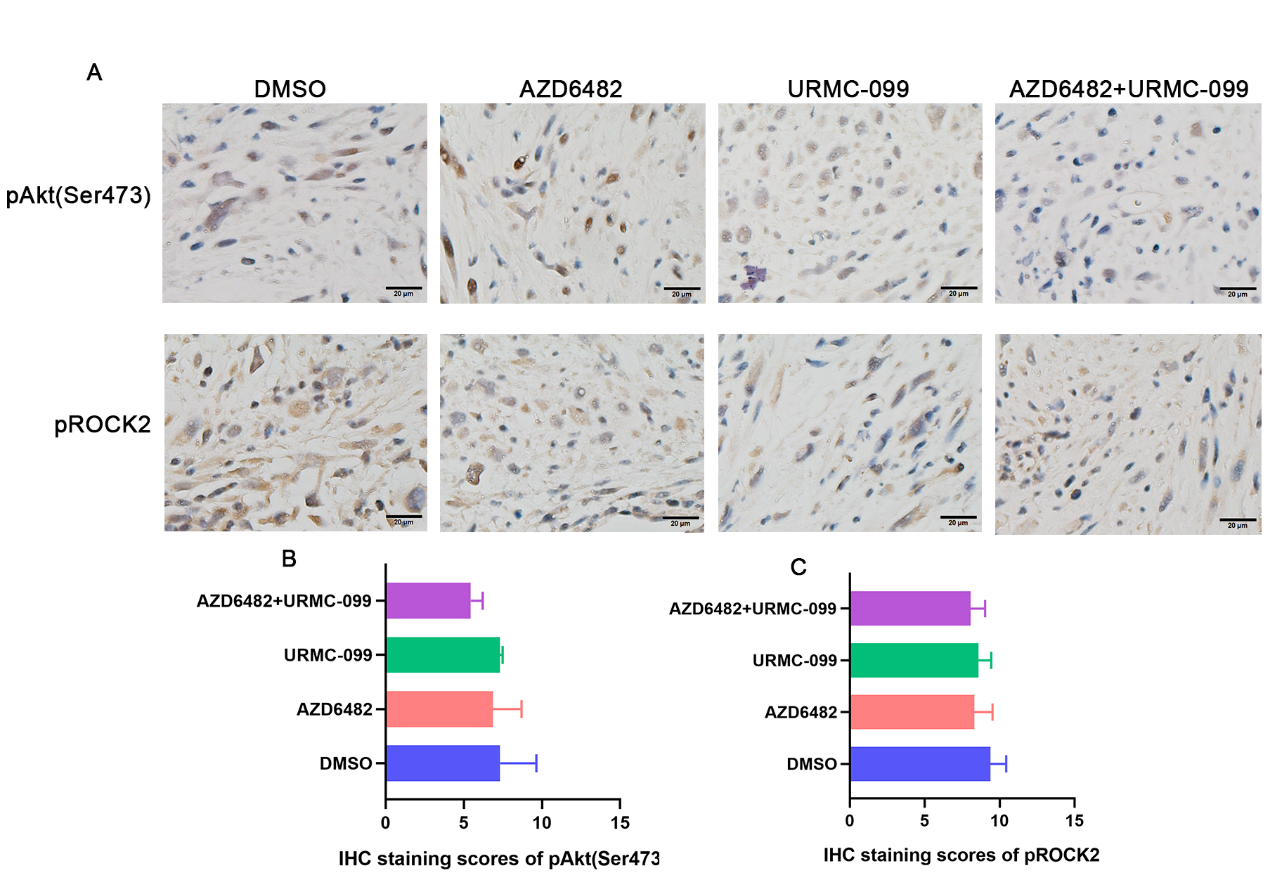


**Supplementary Figure 4: Combination effect of AZD6482 and URMC-099 on the phosphorylation of Akt and ROCK2 in tumor xenografts.** (A) IHC analysis of the phosphorylation of Akt at Ser473 and ROCK2 at Ser1366 in representative sections of tumor xenografts after sacrifice. Bar = 20 μm. (B-C) IHC staining scores of the phosphorylation of Akt and ROCK2 in tumor sections. *P* values were determined by One-way ANOVA and Post Hoc multiple comparison Tukey HSD test.

**Reference**

1. Beaufils F, Cmiljanovic N, Cmiljanovic V, Bohnacker T, Melone A, Marone R, Jackson E, Zhang X, Sele A, Borsari C, Mestan J, Hebeisen P, Hillmann P, Giese B, Zvelebil M, Fabbro D, Williams RL, Rageot D, Wymann MP (2017) 5-(4,6-Dimorpholino-1,3,5-triazin-2-yl)-4-(trifluoromethyl)pyridin-2-amine (PQR309), a Potent, Brain-Penetrant, Orally Bioavailable, Pan-Class I PI3K/mTOR Inhibitor as Clinical Candidate in Oncology. J Med Chem 60:7524-7538. doi:10.1021/acs.jmedchem.7b00930

2. Burger MT, Pecchi S, Wagman A, Ni ZJ, Knapp M, Hendrickson T, Atallah G, Pfister K, Zhang Y, Bartulis S, Frazier K, Ng S, Smith A, Verhagen J, Haznedar J, Huh K, Iwanowicz E, Xin X, Menezes D, Merritt H, Lee I, Wiesmann M, Kaufman S, Crawford K, Chin M, Bussiere D, Shoemaker K, Zaror I, Maira SM, Voliva CF (2011) Identification of NVP-BKM120 as a Potent, Selective, Orally Bioavailable Class I PI3 Kinase Inhibitor for Treating Cancer. ACS Med Chem Lett 2:774-779. doi:10.1021/ml200156t

3. Giordanetto F, Barlaam B, Berglund S, Edman K, Karlsson O, Lindberg J, Nylander S, Inghardt T (2014) Discovery of 9-(1-phenoxyethyl)-2-morpholino-4-oxo-pyrido[1,2-a]pyrimidine-7-carboxamides as oral PI3Kbeta inhibitors, useful as antiplatelet agents. Bioorg Med Chem Lett 24:3936-3943. doi:10.1016/j.bmcl.2014.07.007

4. Goodfellow VS, Loweth CJ, Ravula SB, Wiemann T, Nguyen T, Xu Y, Todd DE, Sheppard D, Pollack S, Polesskaya O, Marker DF, Dewhurst S, Gelbard HA (2013) Discovery, synthesis, and characterization of an orally bioavailable, brain penetrant inhibitor of mixed lineage kinase 3. J Med Chem 56:8032-8048. doi:10.1021/jm401094t

5. Nylander S, Kull B, Bjorkman JA, Ulvinge JC, Oakes N, Emanuelsson BM, Andersson M, Skarby T, Inghardt T, Fjellstrom O, Gustafsson D (2012) Human target validation of phosphoinositide 3-kinase (PI3K)beta: effects on platelets and insulin sensitivity, using AZD6482 a novel PI3Kbeta inhibitor. Journal of thrombosis and haemostasis : JTH 10:2127-2136. doi:10.1111/j.1538-7836.2012.04898.x
